# News and Events of the Week

**Published:** 1909-11-27

**Authors:** 


### News and Events of the Week.

The Royal Society has awarded a special gold medal to Major Ronald Ross, F.R.S., for his researches in connection with malaria.

The King in Council has been pleased to appoint, among others, Miss E. M. N. Williams, M.D., D.P.H., to be a member of the Senate of the University of Durham.

Brigade Surgeon William Pearson Ward, late of the Royal Artillery, a Knight of the Legion of Honour, and a Crimean veteran, who died on September 29, aged eighty-five, left estate valued at £34,487 gross, and £34,397 net.

The Bradshaw lecture of the Royal College of Surgeons will be delivered by Mr. F. Richardson Cross on Friday, December 10, at 5 p.m. The subject of the lecture will be "The Brain Structures Concerned in Vision, and the Visual Field."

Arrangements for the three-day Exhibition of Leadless-glazed China and Earthenware, previously announced to take place in Caxton Hall, Westminster, on November 23, 24, and 25, are now complete. The exhibition will be opened at 12 noon on Tuesday, the 23rd, by the Bishop of Birmingham.

The Council of King's College has appointed Dr. David Waterston, F.R.S. (Ed.), Professor of Anatomy, in succession to Professor Peter Thompson. Dr. Waterston was Lecturer and Senior Demonstrator in Anatomy in the University of Edinburgh. Dr. George C. Low has been elected Lecturer in Parasitology and Medical Entomology. Professor W. D. Halliburton has been elected Dean of the Faculty of Science, Medical Division.

The Lord Lieutenant has appointed Sir William J. Thompson, M.D., to be Registrar-General for Ireland in the room of Sir Robert E. Matheson, LL.D., who vacates office on December 1. Sir William John Thompson was born in 1861, and was educated at Trinity College, Dublin. He is physician-in-ordinary to the Lord Lieutenant, physician to the Jervis Street Hospital, consulting physician to the National Hospital for Consumption, Ireland, and Examiner in Medicine, R.N. Medical Service.

The annual dinner of the Society of Apothecaries was held on November 16. Mr. Warden Norton, in proposing the toast of "The Royal Colleges of Physicians and Surgeons," suggested the advisability of steps being taken whereby the medical students of London might be able to obtain a University degree similar to those granted by the Universities of Scotland and Ireland and the provincial Universities of England. Government should be urged to create a new University, to be called the State University of Medicine, which should give only the degrees of doctor of medicine and surgery. Sir Douglas Powell, President of the Royal College of Physicians, in responding, said that for the last thirty or forty years both the Royal Colleges had been doing their utmost to bring about in some form or other a pass degree for London men, but he thought a State University of Medicine was impossible. The Royal Colleges were at work with the conjoint board considering the question of obtaining a degree for London students, and he was hopeful that some good would be done in that direction by the Royal Commission recently appointed.

The new botanical laboratories at University College will be opened on Friday, December 17, by Dr. D. H. Scott, F.R.S. The Vice-Chancellor (Professor M. J. M. Hill) will preside. Applications for tickets of admission should be made to the Secretary, University College, London, W.C.

The formal organisation by Mr. Nathan Straus of a tuberculosis "preventorium" for children is announced in New York. The idea is to remove young infected children from their homes, and to attack the disease in its earliest stages. If an endowment fund of £200,000 can be raised, and an income of £12,000 be gained from membership fees, 400 will be accommodated free of charge.

Dr. A. Crum Brown, F.R.S., who is well known to Edinburgh graduates, was recently presented by his old pupils with his portrait, painted by Mr. E. A. Walton, R.S.A. Professor James Walker presided, and Sir William Turner, Principal of Edinburgh University, in making the presentation, said that when Dr. Lyon Playfair resigned the chair of chemistry in 1869, Dr. Crum Brown was elected his successor, and he held the position with distinction until he reached the age of seventy last year.

A hundred years ago Napoleon I. offered a prize of 12,000f. for the best essay on croup and its treatment. His little nephew had died of this disease during the previous May. Napoleon's letter empowering the Institute to offer the prize states that the malady had only made its appearance some twenty years previously, but that it had already claimed many victims in Northern Europe, and had invaded France but a few years previously. The prize was divided between two doctors, one from Bremen and one from Geneva.

